# Analysis of Potential Genes and Pathways Involved in the Pathogenesis of Acne by Bioinformatics

**DOI:** 10.1155/2019/3739086

**Published:** 2019-06-09

**Authors:** Biao Chen, Yan Zheng, Yanhua Liang

**Affiliations:** ^1^Department of Dermatology, Guangzhou Women and Children Medical Center, No. 9 Jinsui Road, Guangzhou, Guangdong 510623, China; ^2^Department of Dermatology, The First Affiliated Hospital of Zhengzhou University, 1 Jianshe Eastern Road, Zhengzhou, Henan 450052, China; ^3^Department of Dermatology, Cosmetology and Venereology, Shenzhen Hospital, Southern Medical University, 1333 Xinhu Road, Shenzhen, Guangdong 518101, China

## Abstract

Acne is the eighth most frequent disease worldwide. Inflammatory response runs through all stages of acne. It is complicated and is involved in innate and adaptive immunity. This study aimed to explore the candidate genes and their relative signaling pathways in inflammatory acne using data mining analysis. Microarray data GSE6475 and GSE53795, including 18 acne lesion tissues and 18 matched normal skin tissues, were obtained. Differentially expressed genes (DEGs) were filtered and subjected to functional and pathway enrichment analyses. Protein–protein interaction (PPI) network and module analyses were also performed based on the DEGs. In this work, 154 common DEGs, including 145 upregulated and 9 downregulated, were obtained from two microarray profiles. Gene Ontology and pathway enrichment of DEGs were clustered using significant enrichment analysis. A PPI network containing 110 nodes/DEGs was constructed, and 31 hub genes were obtained. Four modules in the PPI network, which mainly participated in chemokine signaling pathway, cytokine–cytokine receptor interaction, and Fc gamma R-mediated phagocytosis, were extracted. In conclusion, aberrant DEGs and pathways involved in acne pathogenesis were identified using bioinformatic analysis. The DEGs included FPR2, ITGB2, CXCL8, C3AR1, CXCL1, FCER1G, LILRB2, PTPRC, SAA1, CCR2, ICAM1, and FPR1, and the pathways included chemokine signaling pathway, cytokine–cytokine receptor interaction, and Fc gamma R-mediated phagocytosis. This study could serve as a basis for further understanding the pathogenesis and potential therapeutic targets of inflammatory acne.

## 1. Introduction

Acne is a common inflammatory skin disease affecting the pilosebaceous unit. Acne is the eighth most frequent disease worldwide, with a prevalence rate of 94% as evaluated by the Global Burden of Disease Project [1]. Acne can affect the face, neck, chest, and back of both adolescents and adults. The presentation of acne includes comedones, papules, pustules, nodules, cysts, and abscess [2]. In some cases, acne is chronic and persistent, manifesting as onset of lesion in adolescence and persisting in the adult life. The recurrence and persistence of acne usually result in hyperpigmentation and scar, which cause impaired social interaction and serious psychosocial problems, such as anger and depression [2]. Thus, acne has a negative and usual intense impact on the quality of life of patients; for instance, these patients usually have a high unemployment rate [3].

Four critical factors, including follicular colonization by* Propionibacterium acnes (P. acnes)*, increased sebum production, infundibular hyperkeratinization of the pilosebaceous unit, and inflammation, are involved in the pathogenesis of acne. Inflammation plays an important role in the progression of acne lesions [4], and it exists throughout all stages of acne [5]. Microarray is a high-throughput technology used for collecting global gene expression data from recruited samples of different diseases. These microarray data are usually deposited and available in free public websites, such as the NCBI-Gene Expression Omnibus database (NCBI-GEO) (https://www.ncbi.nlm.nih.gov/geo). Trivedi NR et al. performed a microarray analysis of inflammatory acne and found that 211 genes are upregulated in acne lesions; these genes participate in inflammation and extracellular matrix remodeling [6]. Kelhala HL et al. conducted a similar study on early-stage inflammatory acne and found higher levels of Th17 cytokines in lesional skin than in nonlesional skin, indicating that the Th17 pathway is involved in the progression of acne [7]. Integrated bioinformatic analyses of microarray data derived from different studies of acne could help identify the hub genes and further demonstrate their related functions and potential therapeutic targets in inflammatory acne.

In the present study, two public microarray data of GSE6475 and GSE53795 from NCBI-GEO were downloaded. A total of 18 acne lesion (AL) and paired 18 normal skin (NS) data in GSE6475 and GSE53795 were available. DEGs between AL and NS were filtered and obtained using the online tool GEO2R. GO and Kyoto Encyclopedia of Genes and Genomes (KEGG) pathway enrichment analyses of the DEGs were performed using the Database for Annotation, Visualization, and Integrated Discovery (DAVID) (https://david.ncifcrf.gov/). The functions of the DEGs were further assessed by PPI network (http://string-db.org) and modular analyses to identify the hub genes in acne. The study was designed to obtain deep insights into the inflammatory reactions during the pathogenesis of acne.

## 2. Materials and Methods

### 2.1. Microarray Data and Identification of DEGs

GSE6475 and GSE53795 were obtained from NCBI-GEO, a public database of microarray profile and next-generation sequencing, to filter the DEGs between AL and the paired normal control NS. The microarray profile GSE6475 was based on GPL571 platforms (Affymetrix Human Genome U133A 2.0 Array, Palo Alto, CA, USA) and consisted of 6 AL tissues, 6 paired NS tissues, and 6 nonacne NS (Submission date: Dec 07, 2006) [6], although the latter was not included in the present work. The microarray profile GSE53795 was based on GPL570 platforms (Affymetrix Human Genome U133 Plus 2.0 Array, Palo Alto, CA, USA) and included 12 AL tissues and 12 matched NS tissues (Submission date: Jan 03, 2014) [7].

The online tool GEO2R (https://www.ncbi.nlm.nih.gov/geo/geo2r/) was used to analyze the DEGs between AL and NS in the microarray data of GSE6475 and GSE53795, respectively. The adjusted* P*-value and [log⁡FC] were calculated. The Benjamini & Hochberg false discovery rate method was used as a correction factor for the adjusted *P*-value in GEO2R. The statistically significant DEGs were identified according to* P*<0.05 and [log⁡FC] ≥ 1. The common DEGs in GSE6475 and GSE53795 were filtered by the software Functional Enrichment analysis tool (FunRich), which was downloaded from the online website http://www.funrich.org/.

### 2.2. GO and Pathway Enrichment of DEGs in Acne

GO was used to define gene functions in three aspects: molecular function (MF), cellular component (CC), and biological process (BP). DAVID is an online website that provides a comprehensive set of functional annotation tools to understand the biological meaning behind a large list of genes. In the present study, the functional enrichment analyses of the statistically significant DEGs, including GO analysis and KEGG pathway enrichment analysis, were conducted using DAVID, with the cut-off criterion of* P*-value<0.05 and enrichment gene count>2.

### 2.3. Establishment of PPI Network and Modular Analysis

The common DEGs of GSE6475 and GSE53795 were analyzed using the online website STRING (https://string-db.org/, version 11), with 0.700 (moderate confidence) as the minimum required interaction score. Then, the software Cytoscape was used to establish a PPI network. The Network Analyzer in Cytoscape was utilized to calculate node degree. As an App in Cytoscape, CytoHubba was used to identify the hub genes in the PPI network. MCODE was used to perform modular analysis, with the parameters set as follows: a Degree Cutoff=2, Node Score Cutoff=0.2, K-Core=2, and Max. Depth=100. Finally, pathway enrichment analyses of candidate genes in each cluster of PPI network were performed using DAVID (https://david.ncifcrf.gov/).

## 3. Results

### 3.1. Identification of DEGs in Acne

**Figure 1:**
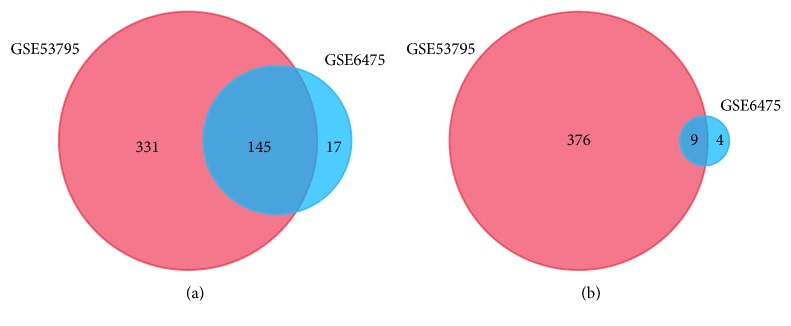
Identification of DEGs from two datasets GSE6475 and GSE53795 using the online tool GEO2R (https://www.ncbi.nlm.nih.gov/geo/geo2r/). The threshold for filtering DEGs is P<0.05 and [log⁡FC] ≥ 1. Different colors indicate different datasets. The cross part represents the common DEGs. (a) Common upregulated DEGs of the two datasets. (b) Common downregulated DEGs of the two datasets.

**Figure 2:**
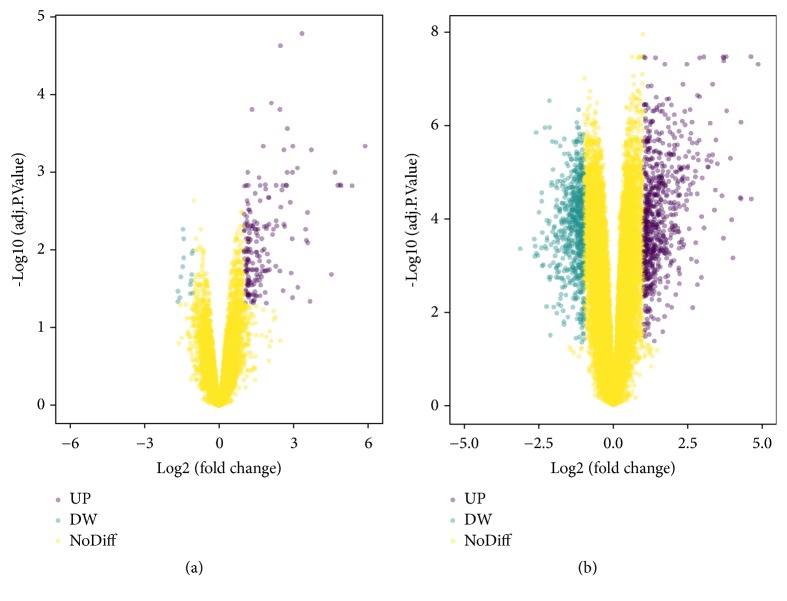
Volcano plot of gene expression in microarray data GSE6475 (a) and GSE53795 (b), with a threshold of P<0.05 and [log⁡FC] ≥ 1 for filtering DEGs. Teal represents downregulated genes, purple represents upregulated genes, and yellow means no significant DEGs. UP, upregulated DEGs; DW, downregulated DEGs; NoDiff, non-differentially expressed genes.

The microarray datasets GSE6475 and GSE53795 were obtained from the public database GEO. We detected 162 upregulated and 13 downregulated genes in GSE6475 and 476 upregulated and 385 downregulated genes in GSE53795, with a threshold of P value<0.05 and fold change (FC) [log⁡FC] ≥ 1. A total of 145 consistently upregulated and 9 concurrently downregulated DEGs were extracted from these two microarray datasets (Figure 1 and Table 1). A total landscape of gene expression in GSE6475 and GSE53795 was presented in a volcano plot (Figure 2), which was produced by the free online website imageGP (http://www.ehbio.com/ImageGP/index.php/Home/Index/Volcanoplot.html).

### 3.2. GO and Pathway Enrichment Analyses of the Upregulated DEGs in Acne

**Figure 3:**
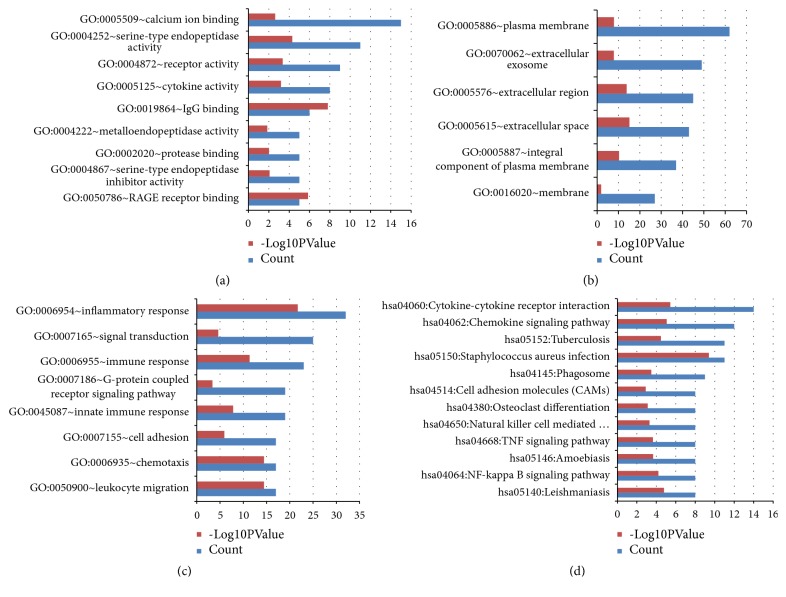
GO and KEGG pathway enrichment analyses of the upregulated DEGs in acne. (a) Significant enriched GO terms in molecular function. (b) Significant enriched GO terms in cellular component. (c) Significant enriched GO terms in biological process. (d) Significant KEGG pathway enrichment for the upregulated DEGs in acne. Count, number of DEGs.

The consistently upregulated DEGs were clustered via the online website DAVID for the functional and KEGG pathway enrichment analyses of DEGs in Acne. The GO analysis of DEGs can be divided into three components: MF, CC, and BP. In terms of MF, the upregulated DEGs were mainly involved in calcium ion binding, serine-type endopeptidase activity, receptor activity, and cytokine activity (Figure 3(a)). As far as CC is concerned, the upregulated DEGs were mainly located in the plasma membrane, extracellular exosome, and extracellular region (Figure 3(b)). As for BP, the upregulated DEGs mainly participated in inflammatory response, signal transduction, immune response, and G-protein coupled receptor signaling pathway (Figure 3(c)). The KEGG pathway of the upregulated DEGs was mainly enriched in cytokine–cytokine receptor interaction, chemokine signaling pathway, phagosome, and NF-kappa B signaling pathway (Figure 3(d), Table S1).

### 3.3. PPI Network of the Common DEGs and Identification of Hub Genes

**Figure 4:**
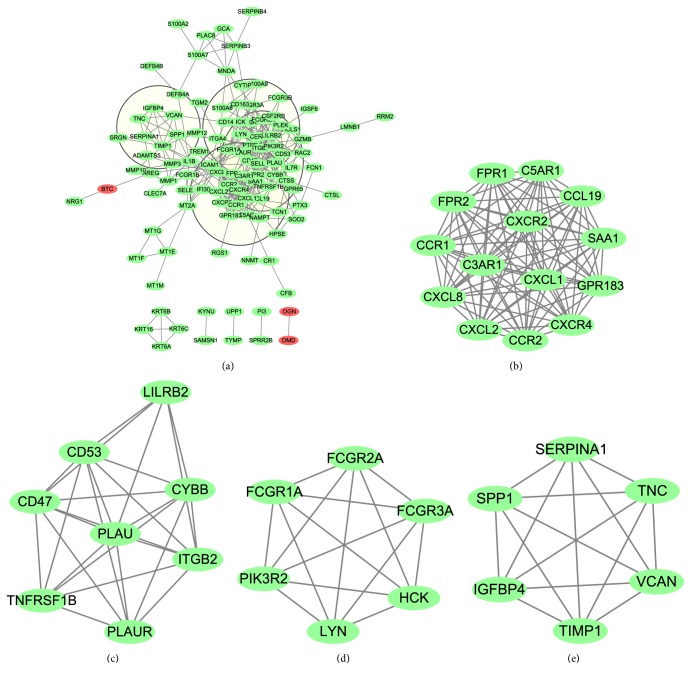
PPI networks of the common DEGs and module analysis. (a) The PPI network contained 110 nodes and 365 edges; four parts of PPI network encompassed by four circles represented four modules filtered by app MCODE in Cytoscape. (b)–(e) represent modules 1–4, respectively, which were extracted from the PPI network. Green represents the upregulated genes, and red represents the downregulated genes.

A PPI network of the common DEGs was constructed using the online website STRING and software Cytoscape. The PPI network contained 110 nodes, including 107 upregulated genes and 3 downregulated genes, and 365 edges; then, 44 of the 154 common DEGs, including 38 upregulated genes and 6 downregulated genes, were excluded in the PPI network (Figure 4(a)). The term “degree” in the PPI network means number of interactions between two genes or two nodes. The hub genes of the PPI network were filtered with a cut-off value of degree≥10. As a result, 31 hub genes were identified (Table S2). The top 12 hub genes included* FPR2*,* ITGB2*,* CXCL8*,* C3AR1*,* CXCL1*,* FCER1G*,* LILRB2*,* PTPRC*,* SAA1*,* CCR2*,* ICAM1*, and* FPR1*, all of which belonged to the common upregulated DEGs (bold in Table 1).

### 3.4. Module Analysis of the PPI Network

With a threshold of MCODE score≥4 and nodes≥6, 4 modules were extracted from the PPI network, including module 1 (Score=14, nodes=14) (Figure 4(b)), module 2 (Score=7.429, nodes=8) (Figure 4(c)), module 3 (Score=6, nodes=6) (Figure 4(d)), and module 4 (Score=6, nodes=6) (Figure 4(e)). All genes of these four modules belonged to the upregulated DEGs. The candidate genes in each module were analyzed using DAVID to produce their relative KEGG pathway enrichment. As shown in Table 2, Module 1 was mainly enriched in chemokine signaling pathway and cytokine–cytokine receptor interaction; KEGG pathway enrichment of Module 2 showed no statistical difference; Module 3 was mainly involved in Fc gamma R-mediated phagocytosis, phagosome, and chemokine signaling pathway; Module 4 participated in ECM–receptor interaction.

## 4. Discussion

Multiple agents are involved in the processes of acne inflammation. In terms of innate immunity,* P. acnes* participates in the inflammation of acne by targeting Toll-like receptors (TLRs) [8]. IL-1*α* is supposed to initiate microcomedos; on the other part,* P. acnes* promotes the production of IL-1*α* by activating the Nod-like receptor 3 inflammasome in monocytes [9, 10]. Other stimuli, such as leukotriens and free fatty acids, can trigger inflammatory reactions in acne [11]. In the adaptive immunity of acne, recruitment of activated Th1 lymphocytes leads to the appearance of early-stage AL [11]. Th17 lymphocytes are also involved in the pathogenesis of acne, presenting as increased expression of IL-17 and IL-22 in peripheral blood mononuclear cells induced by* P. acnes*; IL-17 was detected in the biopsy of AL [12]. Other cytokines or inflammatory markers, such as IL-8, IL-1*β*, beta-defensins 1 and 2, IL-10, TNF-*α*, CXCL-2, and matrix metalloproteinases-1 (MMP-1), MMP-3, and MMP-9, can also be detected [6]. In the present study, we aimed to explore the aberrant DEGs and their relative molecular functions in inflammatory acne through data mining.

Bioinformatic analysis of microarray and/or sequencing data is widely used to explore the aberrant gene expression, potential pathogenesis, and therapeutic targets of various diseases. In the present study, 154 common DEGs, including 145 upregulated and 9 downregulated, were identified in datasets GSE6475 and GSE53795 from GEO. The PPI network contained 31 hub genes, with the addition of the candidate genes in each module, both of them belonged to the upregulated DEGs. Considering the minor number and roles of the downregulated DEGs, we focused on the aberrant upregulated DEGs and their relative molecular mechanisms in acne.

Using the app CytoHubba in Cytoscape, we filtered 31 hub genes in the PPI network. Module 1 contained 14 genes, all of which belonged to hub genes. Among these genes,* SAA1, CCR2*,* C3AR1*,* FPR2*,* FPR1*,* CXCL1*, and* CXCL8* were listed in the top 12 hub genes, and these genes mainly participated in chemokine signaling pathway, cytokine–cytokine receptor interaction, and NOD-like receptor signaling pathway. SAA1, namely, serum amyloid A-1 protein which belongs to the SAA family, is a major and highly conserved acute phase protein. SAA1 and SAA2 (another member of SAA family) contribute to inflammatory skin diseases such as acne. Su Q et al. showed that glucocorticoids in combination with* P. acnes* increase SAA1 and SAA2 expression, whereas glucocorticoids promote SAA1 production by combining with TNF, indicating that glucocorticoids induce SAA1 expression under infectious and sterile inflammatory circumstances, promoting the progress of cutaneous inflammation [13]. CCR2 (C-C Motif Chemokine Receptor 2) is a receptor for chemokines CCL2, CCL7, and CCL13 and for defensins DEFB106A/DEFB106B, which are antimicrobial and cytotoxic peptides produced by neutrophils. As antimicrobial peptides expressed by skin, defensins serve as part of the innate immunity in response to cutaneous pathogens. Increased expression of DEFB1, DEFB2, and DEFB4 is observed in acne lesions, indicating that defensins play a role in host defense mechanism against microbial pathogens in acne [6]. CCR2 specifically mediates monocyte infiltration by combining with monocyte chemoattractant protein-1. C3AR1 and C5AR1, C5AR2 are the receptors for C3a and C5a, respectively, which form the central part of the complement system, and the process manages cellular response to inflammation [14]. The combination of C3a and C3AR1 leads to the production of superoxide anion, release of granule enzymes, and bacterial opsonization. FPR1 and FPR2, members of the formyl peptide receptor family, are Gi-protein-coupled receptors that are mainly expressed in leukocytes and participate in antibacterial and inflammatory processes. CXCL1 and CXCL8 are members of the CXC chemokine family functioning in chemokine activity and signaling receptor binding. CXCL1 participates in chemotactic activity for neutrophils and functions in inflammation. As a paralog of CXCL1, CXCL2, which participates in inflammatory processes, is upregulated in AL [6]. CXCL8, namely, IL8, has a chemotactic capacity for neutrophils, basophils, and T-cells, as well as participating in neutrophil activation. As an antimicrobial and anti-inflammatory medicine, tetracyclines suppress the ATP gamma S-induced release of proinflammatory mediators, including CXCL1 and CXCL8, by HMEC-1 cells and primary human dermal microvascular endothelial cells, to improve cutaneous inflammatory diseases [15]. Askari N et al. found that proinflammatory cytokines, including IL-1*β*, IL-8, IL-12, and RANTES, positively correlate with acne under mustard gas-exposed conditions [16].

Genes of Module 3 included* PIK3R2*,* FCGR1A*,* FCGR2A*,* LYN*,* HCK*, and* FCGR3A*, which are mainly involved in Fc gamma R-mediated phagocytosis. Phosphatidylinositol 3-kinase (PI3K) is a lipid kinase containing a regulatory and a catalytic subunit. PIK3R2 is a regulatory subunit of PI3K, which phosphorylates phosphatidylinositol 4,5-bisphosphate to produce phosphatidylinositol 3,4,5-trisphosphate and recruits PH domain-containing proteins, such as Akt1, to the membrane. Activation of the PI3K-Akt pathway plays a role in cell survival, proliferation, and motility [17]. Shi G et al. showed that FoxO1 regulated by the PI3K-Akt pathway mediates keratinocyte differentiation, which could be involved in acne pathogenesis and serve as a potential treatment target [18]. Suppression of PI3K/Akt signaling inhibits lipogenesis induced by TNF-*α*, which is associated with acne development [19]. Activation of the PI3K/Akt/mTOR pathway mediated by insulin in sebocytes leads to high protein/lipid synthesis, cell proliferation, and inflammation [20].

Genes of Module 4 included* SERPINA1*,* VCAN*,* IGFBP4*,* SPP1*,* TNC*, and* TIMP1*, in which* TNC* and* SPP1* were clustered and participated in ECM–receptor interaction. The extracellular matrix (ECM) consists of a variety of macromolecules. The combination of ECM and specific transmembrane molecules (mainly integrins) of cells plays a role in cellular activities, including proliferation, apoptosis, differentiation, adhesion, and migration. Both TNC and SPP1 are matricellular proteins that are upregulated in active tuberculosis (TB) relative to healthy controls or latent TB; OPN (i.e., SPP1) and TNC may function as reliable biomarkers for monitoring TB activity [21]. TNC facilitates sterile inflammation by activating TLR4; TNC could serve as an endogenous protein that triggers inflammation by recognizing TLRs [22]. Both TNC and lipopolysaccharide stimulate TLR4 and activate different signaling pathways, leading to divergent phenotypes of macrophages [23]. OPN is a Th2 inflammation-related protein. In allergic rhinitis, upregulated SPP1 expression induced by leptin facilitates Th2 inflammation, and this process is mediated by *α*4 integrin and PI3K/Akt pathway [24]. Tear OPN expression in patients with perennial allergic conjunctivitis (AC) is higher than that in patients with seasonal AC due to the pollen season and in healthy controls; therefore, tear OPN expression possibly functions in local Th2/17 cytokine production and positively correlates with disease severity [25]. However, the roles of TNC and SPP1 in acne have not been reported until now.

In conclusion, the present study exhibits the global profile of DEGs and relative signaling pathways that might participate in the initiation and development of acne mechanically. In the pathogenesis of acne, the possible crucial genes are* FPR2*,* ITGB2*,* CXCL8*,* C3AR1*,* CXCL1*,* FCER1G*,* LILRB2*,* PTPRC*,* SAA1*,* CCR2*,* ICAM1*, and* FPR1*, and the possible important pathways are chemokine signaling pathway, cytokine–cytokine receptor interaction, and Fc gamma R-mediated phagocytosis. These results could help elucidate the molecular mechanism underlying acne pathogenesis and provide potential targets for acne therapy. Further investigations are needed to confirm our putative finding.

## Figures and Tables

**Table 1:** 154 DEGs were extracted from 2 microarray data GSE6475 and GSE53795, including 145 up-regulated genes and 9 down-regulated genes in acne lesions, comparing to normal skin of acne patients.

DEGs	Gene symbol
Up-regulated genes	*DEFB4B, DEFB4A, SERPINB4, MMP1, * ***CXCL8*** *, MMP3, * ***CXCL1*** *, APOBEC3A, S100A12, PI3, SPP1, AKR1B10, BCL2A1, CXCL2, MMP12, SERPINB3, * ***LILRB2*** *, SPRR2C, * ***FPR1*** *, FCGR1B, PLA2G2A, PTX3, TCN1, SERPINA1, SELE, MT1M, GZMB, CCR1, C5AR1, SOD2, MMP10, CCL19, IL36G, S100A9, SAMSN1, FCGR1CP, FCGR1A, SERPINB1, FCGR3B, FCGR3A, FCN1, CLEC7A, SELL, TREM1, PLAUR, * ***FCER1G*** *, KYNU, SPRR2B, GREM1, NAMPT, CFB, NNMT, CD163, IGSF6, IL7R, PLAC8, * ***FPR2*** *, IFI30, PIK3R2, RGS1, SAA2, * ***SAA1*** *, CXCR4, RHCG, PLEK, ADGRE2, SLC6A14, SRGN, LYN, GPR65, FCGR2A, IL1B, S100A7, GPR183, * ***C3AR1*** *, STEAP1, SLC16A3, CSF3R, TIMP1, CR1, KRT16, SNX10, COTL1, CD53, HCK, SLC39A14, SLC7A11, KLK13, MT1G, CYBB, UPP1, PDPN, CXCR2, KIAA0226L, CSF2RB, TNC, CD14, MT1F, TNFRSF1B, S100A2, THEMIS2, * ***PTPRC*** *, NRG1, MNDA, ADAMTS5, TYMP, * ***ICAM1*** *, CTSL, CYTIP, IGFBP4, HCLS1, CEMIP, PLSCR1, HPSE, MT1E, MT2A, SLAMF8, RRM2, BASP1, CD47, RAC2, PLAU, * ***ITGB2*** *, HS3ST1, VCAN, CDH3, * ***CCR2*** *, CLEC4A, CTSS, TTC39A, TGM2, ITGA4, SLC20A1, DMXL2, S100A8, GCA, LMNB1, PILRA, TNFRSF6B, MPZL2, KRT6A, KRT6B, KRT6C, AREG, ARNTL2*

Down-regulated genes	*HLF, ID4, ZBTB16, TSPAN8, SGCG, LRRC17, OGN, OMD, BTC*

*Abbreviations*: DEGs, differentially expressed genes.

**Table 2:** KEGG pathway enrichment analysis of genes of Module 1~4 in PPI network.

Term	Description	Count	p-Value	Genes
*Module 1*				
hsa04062	Chemokine signaling pathway	8	2.85E-09	*CXCL1, CXCR4, CCR1, CCR2, CXCL2, CXCL8, CCL19, CXCR2*
hsa04060	Cytokine-cytokine receptor interaction	8	1.84E-08	*CXCL1, CXCR4, CCR1, CCR2, CXCL2, CXCL8, CCL19, CXCR2*
hsa05150	Staphylococcus aureus infection	4	7.22E-05	*C3AR1, C5AR1, FPR1, FPR2*
hsa04080	Neuroactive ligand-receptor interaction	4	0.008378	*C3AR1, C5AR1, FPR1, FPR2*
hsa05134	Legionellosis	3	0.003179	*CXCL1, CXCL2, CXCL8*
hsa04621	NOD-like receptor signaling pathway	3	0.003416	*CXCL1, CXCL2, CXCL8*
hsa05132	Salmonella infection	3	0.007372	*CXCL1, CXCL2, CXCL8*
*Module 2*				
hsa04610	Complement and coagulation cascades	2	0.058715	*PLAU, PLAUR*
*Module 3*				
hsa04666	Fc gamma R-mediated phagocytosis	5	1.03E-07	*LYN, FCGR1A, HCK, FCGR2A, PIK3R2*
hsa04380	Osteoclast differentiation	4	6.56E-05	*FCGR1A, FCGR2A, FCGR3A, PIK3R2*
hsa05150	Staphylococcus aureus infection	3	5.96E-04	*FCGR1A, FCGR2A, FCGR3A*
hsa05140	Leishmaniasis	3	0.00103	*FCGR1A, FCGR2A, FCGR3A*
hsa04611	Platelet activation	3	0.003414	*LYN, FCGR2A, PIK3R2*
hsa05322	Systemic lupus erythematosus	3	0.003624	*FCGR1A, FCGR2A, FCGR3A*
hsa04145	Phagosome	3	0.004524	*FCGR1A, FCGR2A, FCGR3A*
hsa05152	Tuberculosis	3	0.006255	*FCGR1A, FCGR2A, FCGR3A*
hsa04062	Chemokine signaling pathway	3	0.006891	*LYN, HCK, PIK3R2*
hsa04664	Fc epsilon RI signaling pathway	2	0.048472	*LYN, PIK3R2*
hsa04662	B cell receptor signaling pathway	2	0.049171	*LYN, PIK3R2*
*Module 4*				
hsa04512	ECM-receptor interaction	2	0.049648	*TNC, SPP1*

*Notes*: Count, the number of DEGs.

*Abbreviations*: DEGs, differentially expressed genes; KEGG, Kyoto Encyclopedia of Genes and Genomes; PPI, protein–protein interaction.

## Data Availability

The data used to support the findings of this study are included within the article and the supplementary information files.

## Supplementary Materials

Supplementary Materials Table S1: KEGG pathway enrichment analysis for the common upregulated DEGs of GSE6475 and GSE53795 in acne. Table S2: the hub genes in the PPI network (degree ≥ 10).
