# Supplementary material for: Analysis of Potential Genes and Pathways Involved in the Pathogenesis of Acne by Bioinformatics
Source: Biomed Res Int. 2019 Jun 9;2019:3739086. doi: 10.1155/2019/3739086 (PMC6590534; doi:10.1155/2019/3739086)
Supplement: Supplementary Materials — Table S1: KEGG pathway enrichment analysis for the common upregulated DEGs of GSE6475 and GSE53795 in acne. Table S2: the hub genes in the PPI network (degree ≥ 10). [file 3739086.f1.pdf]

Supplementary Materials

Table S1. KEGG pathway enrichment analysis for the common upregulated DEGs of GSE6475 and GSE53795 in acne.

Term	Description	Count	*p*-Value	Genes
hsa04060	Cytokine-cytokine receptor interaction	14	3.70E-06	*TNFRSF6B, CXCL1, CCR1, CXCL2, CCL19, CXCL8, CXCR2, IL7R, TNFRSF1B, CXCR4, CCR2, CSF3R, IL1B, CSF2RB*
hsa04062	Chemokine signaling pathway	12	8.66E-06	*CXCL1, RAC2, LYN, CXCR4, CCR1, HCK, CCR2, CXCL2, CXCL8, CCL19, CXCR2, PIK3R2*
hsa05150	Staphylococcus aureus infection	11	4.03E-10	*ICAM1, C3AR1, C5AR1, CFB, FCGR1A, FPR1, ITGB2, FCGR2A, FPR2, FCGR3A, FCGR3B*
hsa05152	Tuberculosis	11	3.42E-05	*CR1, FCGR1A, FCER1G, IL1B, ITGB2, FCGR2A, CLEC7A, CTSS, FCGR3A, FCGR3B, CD14*
hsa04145	Phagosome	9	3.26E-04	*CTSL, FCGR1A, ITGB2, FCGR2A, CLEC7A, CTSS, FCGR3A, FCGR3B, CD14*
hsa05140	Leishmaniasis	8	1.61E-05	*CR1, FCGR1A, IL1B, ITGB2, FCGR2A, ITGA4, FCGR3A, FCGR3B*
hsa04064	NF-kappa B signaling pathway	8	6.10E-05	*ICAM1, LYN, BCL2A1, CXCL8, CCL19, IL1B, CD14, PLAU*
hsa05146	Amoebiasis	8	2.13E-04	*CXCL8, SERPINB1, IL1B, SERPINB4, ITGB2, SERPINB3, CD14, PIK3R2*
hsa04668	TNF signaling pathway	8	2.26E-04	*CXCL1, ICAM1, TNFRSF1B, CXCL2, IL1B, MMP3, SELE, PIK3R2*
hsa04650	Natural killer cell mediated cytotoxicity	8	5.05E-04	*ICAM1, RAC2, FCER1G, GZMB, ITGB2, FCGR3A, FCGR3B, PIK3R2*
hsa04380	Osteoclast differentiation	8	7.74E-04	*LILRB2, CYBB, FCGR1A, IL1B, FCGR2A, FCGR3A, FCGR3B, PIK3R2*
hsa04514	Cell adhesion molecules (CAMs)	8	0.001245	*ICAM1, PTPRC, SELL, VCAN, ITGB2, ITGA4, CDH3, SELE*
hsa05134	Legionellosis	7	3.39E-05	*CXCL1, CR1, CXCL2, CXCL8, IL1B, ITGB2, CD14*
hsa04610	Complement and coagulation cascades	7	1.37E-04	*C3AR1, CR1, C5AR1, CFB, SERPINA1, PLAU, PLAUR*
hsa04666	Fc gamma R-mediated phagocytosis	7	4.04E-04	*PTPRC, RAC2, LYN, FCGR1A, HCK, FCGR2A, PIK3R2*
hsa04640	Hematopoietic cell lineage	7	4.89E-04	*CR1, FCGR1A, IL1B, CSF3R, ITGA4, IL7R, CD14*
hsa05323	Rheumatoid arthritis	7	5.20E-04	*CTSL, ICAM1, CXCL8, IL1B, ITGB2, MMP3, MMP1*

hsa05202	Transcriptional misregulation in cancer	7	0.012794	*FCGR1A, BCL2A1, CXCL8, GZMB, MMP3, CD14, PLAU*
hsa04978	Mineral absorption	6	1.43E-04	*MT1M, MT2A, MT1E, STEAP1, MT1G, MT1F*
hsa05144	Malaria	6	2.40E-04	*ICAM1, CR1, CXCL8, IL1B, ITGB2, SELE*
hsa04670	Leukocyte transendothelial migration	6	0.010633	*ICAM1, RAC2, CXCR4, ITGB2, ITGA4, PIK3R2*
hsa05132	Salmonella infection	5	0.015572	*CXCL1, CXCL2, CXCL8, IL1B, CD14*
hsa04620	Toll-like receptor signaling pathway	5	0.034504	*CXCL8, IL1B, CD14, SPP1, PIK3R2*
hsa04621	NOD-like receptor signaling pathway	4	0.027007	*CXCL1, CXCL2, CXCL8, IL1B*
hsa04664	Fc epsilon RI signaling pathway	4	0.044255	*RAC2, LYN, FCER1G, PIK3R2*

Notes: Count, the number of DEGs.

Abbreviations: KEGG, Kyoto Encyclopedia of Genes and Genomes; DEGs, differentially expressed genes.

Table S2: The hub genes in the PPI network (degree ⩾ 10)

ID	Degree	ID	Degree	ID	Degree	ID	Degree
FPR2	24	*ICAM1*	16	*C5AR1*	14	*HCK*	12
CXCL8	23	*FPR1*	16	*GPR183*	14	*PIK3R2*	12
ITGB2	23	*CCR2*	16	*CXCR2*	13	*LYN*	11
C3AR1	21	*SAA1*	16	*CYBB*	13	*PLAU*	10
CXCL1	19	*CXCR4*	15	*CCR1*	13	*PLAUR*	10
PTPRC	17	*CXCL2*	14	*FCGR1A*	13	*FCGR2A*	10
LILRB2	17	*CCL19*	14	*TIMP1*	12	*CD47*	10
FCER1G	17	*CD53*	14	*IL1B*	12		

Note: Degree, the number of interaction between two genes in PPI network.

Abbreviations: PPI, protein–protein interaction.
